# Role of different anti-seizure medications on carotid intima–media thickness: A systematic review and meta-analysis

**DOI:** 10.1097/MD.0000000000045792

**Published:** 2025-11-07

**Authors:** Afshan Davari, Amir Reza Bahadori, Ali Mohammadi-Asl, Rasa Zafari, Mehrdad Sheikhvatan, Sara Ranji, Sajad Shafiee, Abbas Tafakhori

**Affiliations:** a Iranian Center of Neurological Research, Neuroscience Institute, Tehran University of Medical Sciences, Tehran, Iran; b Neurology Department, Medical Colleges, Tehran University of Medical Sciences, Tehran, Iran; c Neurology Department, School of Medicine, Tehran University of Medical Sciences, Tehran, Iran; d Medical Biology and Genetics Department, Okan University, Istanbul, Turkey; e Department of Neurology, Heidelberg University, Heidelberg, Germany; f Neurosurgery Department, Mazandaran University of Medical Sciences, Sari, Iran.

**Keywords:** anti-seizure medication, carotid intima–media thickness, CIMT, epilepsy

## Abstract

**Background:**

Various types of anti-seizure medication (ASMs) may have an impact on the cardiac health of patients who have epilepsy. One of the most important noninvasive predictive factors related to cardiovascular disease is carotid intima–media thickness (CIMT). The objective of this systematic review and meta-analysis is to determine the effect of both mono- and poly-therapy with ASMs on CIMT.

**Methods:**

Four databases (PubMed, Scopus, Web of Science, and Embase) were searched for records. Studies that measured the effect of ASMs on CIMT were eligible to be included. The case and control data of studies were extracted. Also, the quality assessment of each included study was assessed by the risk of bias in non-randomized studies of interventions (ROBINS-I) checklist. Additionally, the random-effect model analysis was performed by the *Comprehensive Meta-Analysis Software* (CMA) version 3.0.

**Results:**

Twenty-nine studies were included in the systematic review, and 15 studies were eligible to perform meta-analysis. Among both poly-therapy, and monotherapy with valproate sodium, carbamazepine, levetiracetam, and phenytoin, CIMT had significantly higher thickness compared to the control group (SMD: 1.82, 1.18, 1.33, 1.83, and 1.15 with 95%CI: [0.34, 3.3], [0.67, 1.7], [0.38, 2.27], [0.1, 3.56], and [−0.49, 2.8], respectively) (*P*-value: .001, .001, .001, .001, and .007, respectively).

**Conclusion:**

The potential risk of cardiovascular diseases related to CIMT may be elevated by using both mono- and polytherapy with valproate sodium, carbamazepine, levetiracetam, and phenytoin.

## 1. Introduction

Epilepsy is a chronic neurological disorder defined by recurrent seizures, affecting 50 million individuals worldwide.^[1]^ The management of epilepsy in most cases involves long-term treatment with anti-seizure medications (ASMs), ketogenic diet, neuromodulation, and in a few cases, surgical interventions.^[2]^ Although these treatments are effective in controlling seizures, they may have some potential side effects on cardiovascular health Phenytoin and carbamazepine have been linked to increased risk of atherosclerosis by elevating lipid profile and promoting oxidative stress.^[3]^ Valproic acid may also contribute to metabolic syndrome that causes increased cardiovascular risk.^[4]^ Carotid intima–media thickness (CIMT) is a noninvasive marker of atherosclerosis and an important predictive factor of cardiovascular events, such as myocardial infarction, sudden death, and stroke. Also, elevated CIMT has been associated with a higher risk of cardiovascular disease, and it is broadly used in clinical and research settings to evaluate subclinical atherosclerosis.^[5,6]^ Emerging evidence suggests that some ASMs like Valproic acid or Phenytoin may influence cardiovascular health, including changes in lipid profiles, blood pressure, and CIMT.^[7]^ The link between ASMs and CIMT involves several mechanisms. ASMs cause weight gain and dyslipidemia that leads to atherosclerosis. Also, Phenytoin interfere with folate metabolism that leads to elevated homocysteine that is a recognized risk factor for atherosclerosis and cardiovascular disease. Furthermore, Phenytoin and Valproic acid can cause oxidative stress and inflammation, leading to endothelial dysfunction and thickening of the arterial wall.^[8]^ As well, certain ASMs have been linked to adverse lipid changes and increased risk of atherosclerosis, while others may have protection against cardiovascular disease.^[9]^ Understanding the impact of epilepsy treatments on CIMT is critical for improvement of treatment strategies and decreasing cardiovascular risk in individuals with epilepsy. Despite the growing number of research papers on the cardiovascular effects of epilepsy treatments, the association between these treatments and CIMT is controversial.^[7,10]^ Previous studies have reported inconsistent results, and there has been no comprehensive synthesis of the evidence.^[10]^ A systematic review and meta-analysis is necessary to merge existing data, evaluate the strength of the available evidence, and identify potential areas for future research. The primary objective of this systematic review and meta-analysis is to evaluate the association between current epilepsy treatments and CIMT. Specifically, we aim to assess the impact of different antiepileptic drugs on CIMT. Compare the effects of various medications on CIMT to identify any significant differences. Investigate potential sources of heterogeneity in the findings, such as differences in study design, population characteristics, and treatment duration. By investigating this title, this review will provide valuable insights into the cardiovascular complications of epilepsy treatments and improve clinical practice and decision-making.

## 2. Materials and methods

The aim of current systematic review and meta-analysis is to explore the association between available medical treatments of epilepsy and CIMT. Our methodology adheres to the PRISMA (Preferred Reporting Items for Systematic Reviews and Meta-analyses) guidelines^[11]^ and also was registered in the International Prospective Register of Systematic Reviews (PROSPERO) under the ID number: CRD42024564940.

### 2.1. Literature search strategy

A comprehensive literature search was conducted up to May 14, 2024, to retrieve relevant articles from following databases: PubMed, Scopus, Embase, and Web of Science (WOS). The search strategy consisted of 2 main subgroups of keywords and of Medical Subject Headings (MeSH). One subgroup consisted of terms related to Epilepsy, the other one included terms related to CIMT. The subgroups were combined using the “AND” operator, and no restrictions were applied regarding the date, or publication type. The search strategy was modified according to the search query format of each database. Full search query of each database is detailed in Supplementary File A (Supplemental Digital Content, https://links.lww.com/MD/Q573).

### 2.2. Study selection and data extraction

The selection process involved 2 stages: screening: titles and abstracts of identified studies were independently screened by 2 reviewers (AM and RZ). Studies were included if they enrolled patients diagnosed with epilepsy, reported CIMT measurements, were original research articles (observational or interventional) published in peer-reviewed journals, and provided clear information on sample size and follow-up duration. Studies that did not meet the inclusion criteria were excluded. Studies were excluded if they focused on diet or interventions unrelated to epilepsy, were reviews, case reports, editorials, or abstracts, or included participants with comorbid conditions that could independently affect CIMT (e.g., cardiovascular disease or diabetes). The full texts of potentially relevant studies were independently assessed by the same reviewers (AM and RZ). Discrepancies were resolved through discussion or by consulting a third reviewer (AD).

Furthermore, the data extraction sheet was designed by ARB and the Excel sheet included the following items in 4 sets: study characteristics (i.e., authors, location, year, number of participants and type of study); patient-specific factors (i.e., the eligibility criteria for included cases); study design and intervention (i.e., type of epilepsy, type of intervention and time of follow-up); and CIMT size. Moreover, 2 reviewers did the risk of bias assessment using a tool for assessing risk of bias in non-randomized studies of interventions (ROBINS-I).^[12]^ A third author was involved in the process in case of disagreement.

## 3. Statistical analysis

We used *Comprehensive meta-analysis (CMA*) software (BioStat Inc., Englewood), developed version 3.0, for our data analysis. Results were reported as pooled standard error with a 95% confidence interval, visualized in a forest plot. We evaluated heterogeneity among the eligible studies using the *I*^2^ statistic and used the random effects model when significant heterogeneity was detected (*I*^2^ > 50%). To further explore potential sources of heterogeneity, meta-regression analyses were subsequently performed using study-level. We also conducted the Egger test to assess publication bias across the included studies. For ASMs where funnel plot asymmetry was detected, we applied the Duval and Tweedie Trim and Fill method to estimate and adjust for potentially missing studies. Finally, we performed a sensitivity analysis by excluding 1 study at a time and repeating the meta-analysis to ensure the reliability of our findings.

## 4. Result

### 4.1. Study feature

**Figure 1.**
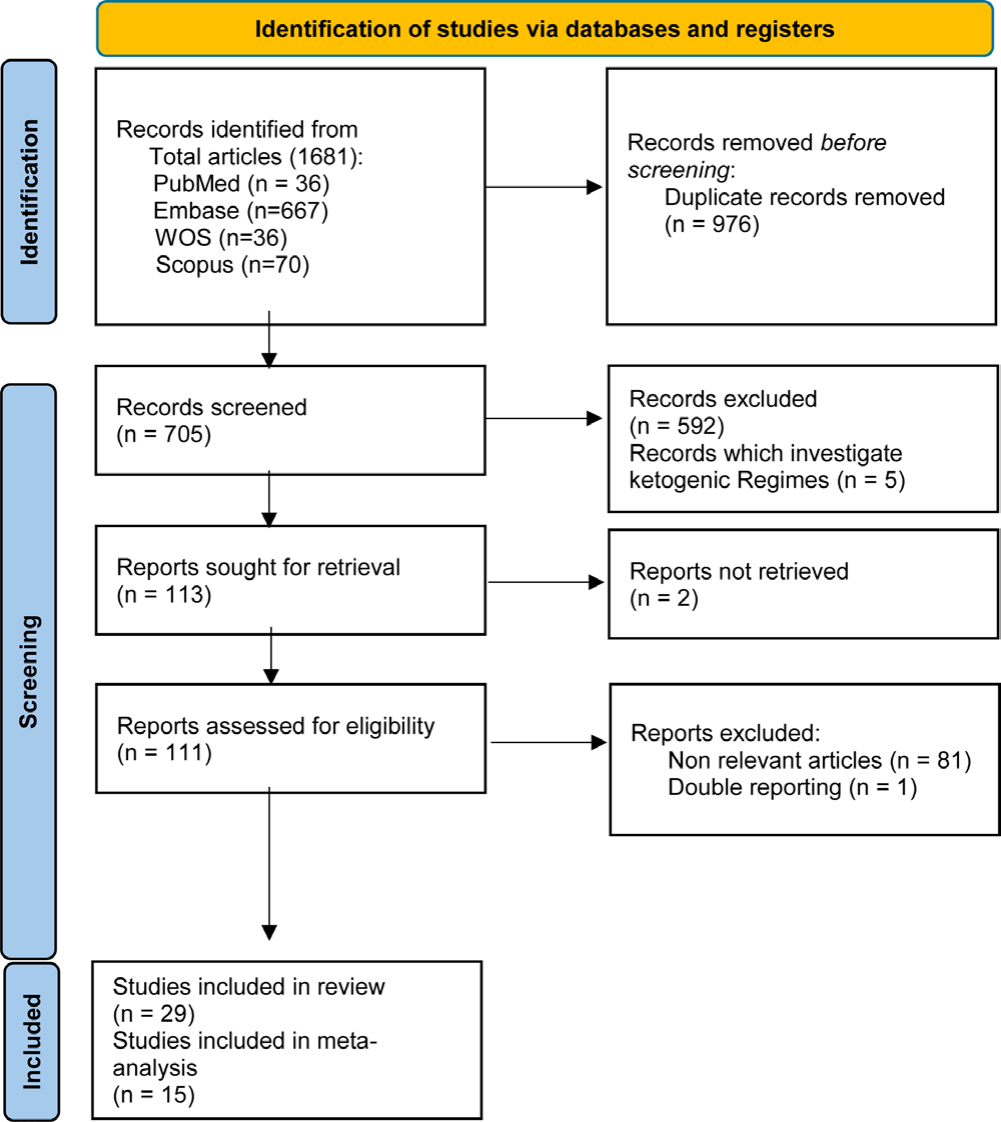
PRISMA flow diagram of effect of anti-seizure medications on size of carotid intima–media thickness. PRISMA = preferred reporting items for systematic reviews and meta-analyses.

We carried out this survey to compare the effects of different types of ASM on CIMT in individuals with epilepsy. 1681 Records were identified according to relevant keywords from databases. After excluding 976 duplicated articles, 705 records were checked based on title and abstract. After full text screening 29 articles eligible for systematic review. It should be mentioned that, 7 studies were excluded owing to data reporting bias,^[6,13–18]^ 7 studies did not match the type of ASM with CIMT^[19–25]^ (Fig. 1). Therefore, in total, 15 studies were included in our meta-analysis. The characteristics of each study are illustrated in Table 1. Each study was evaluated for risk of bias by 2 researchers using the ROBINS-Ⅰ.^[12]^ According to the ASM therapy, remaining studies were divided into 5 subgroups (valproate sodium [VPA], n = 9; carbamazepine [CBZ], n = 8; levetiracetam [LEV], n = 4; phenytoin [PHN], n = 4; and poly-therapy, n = 5). We ran 5 meta-analyses in the mentioned subgroups.

**Table 1:** The characteristics of studies assessed.

Author, year	Country	Type of study	Type of ASM	No. of patients/male	Age of patients (year)	No. of controls/male	Age of controls (yr)	Duration of use of drug
Hamed et al, 2007	Egypt	Case-control study	Valproate	35/_	_	60/38	30.24 ± 9.70	_
			Carbamazepine	85/_	_			_
			Poly-therapy	20/_	_			_
Erdemir et al, 2009	Turkey	Cross-sectional study	Valproate	44/26	11.159 ± 3.275	40/21	11.688 ± 3.129	At least 1 yr
Chuang et al, 2012	Taiwan	Cross-sectional study	Valproate	54/32	33.8 ± 11.2	60/30	34.5 ± 10.5	8.7 ± 5.2 yr
			Carbamazepine	41–20	34.1 ± 9.5			13.4 ± 6.3 yr
			Phenytoin	39/22	37.8 ± 8.9			10.7 ± 8.2 yr
Yis et al, 2012	Turkey	Case-control study	Carbamazepine	21/10	7.42 ± 1.39	22/10	7.72 ± 2.14	8.2 ± 4.2 mo
Sankhyan et al, 2013	India	Cross-sectional study	Carbamazepine	28/20	9.1 ± 2	28/20	9.3 ± 2	30.8 ± 13.2 mo
			Phenytoin	30/18	9.4 ± 2	30/20		29.5 ± 13.6 mo
El-Falahaty et al, 2015	Egypt	Cross-sectional study	Valproate	20/12	15.6 ± 7.3	34/18	11.6 ± 1.9	5.5 + 2.0 yr
			Carbamazepine	14/8	15.6 ± 3.95			8.5 + 4.2 yr
			Levetiracetam	12/10	13.6 ± 6.7			2.2 yr
Luo et al, 2015	China	Case-control study	Valproate	30/15	21.9 ± 3.68	33/16	22 ± 3.562	more than 1 yr
Bano et al, 2017	Pakistan	Cross-sectional study	Carbamazepine	33/18	34 ± 9.5	100/54	34 ± 9.5	<1 yr
Ksoo et al, 2017	India	Observational prospective study	Valproate	55/-	1–18*	67/-	1–18*	12 mo
			Carbamazepine	66/-				
			Phenytoin	4/-				
Calik et al, 2018	Turkey	Case-control study	Poly-therapy	30/-	_	34/16	8.58 ± 4.49	3.23 ± 2.45 yr
Kolekar et al, 2021	India	Case-control study	Valproate	30/21	6.58†	42/27	_	At least 6 mo
			Levetiracetam	3/1	6†			At least 6 mo
			Phenytoin	9/5	7†			At least 6 mo
Lima et al, 2021	Iran	Case-control study	Poly-therapy	20/10	33.0 ± 6.1	20/10	32.9 ± 6.1	At least 6 yr
Mahmoud et al, 2022	Egypt	Case-control study	Valproate	53/36	3.4 ± 2.6	53/34	3.9 ± 2.3	For over 6 mo
			Levetiracetam	53/29	4.2 ± 2.1			For over 6 mo
			Poly-therapy	53/35	3.4 ± 1.6			For over 6 mo
Mahrous et al, 2022	Egypt	Cross-sectional study	Poly-therapy	20/12	6.5†	25/13	6†	3 yr
Nasef et al, 2023	Egypt	Case-control study	Valproate	32/25	7.16†	60/32	6.08†	At least 6 mo
			Carbamazepine	11/5	6.83†			At least 6 mo
			Levetiracetam	17/5	6.5†			At least 6 mo

* Data given as range.

† Data given as median.

### 4.2. Study analysis

**Figure 2.**
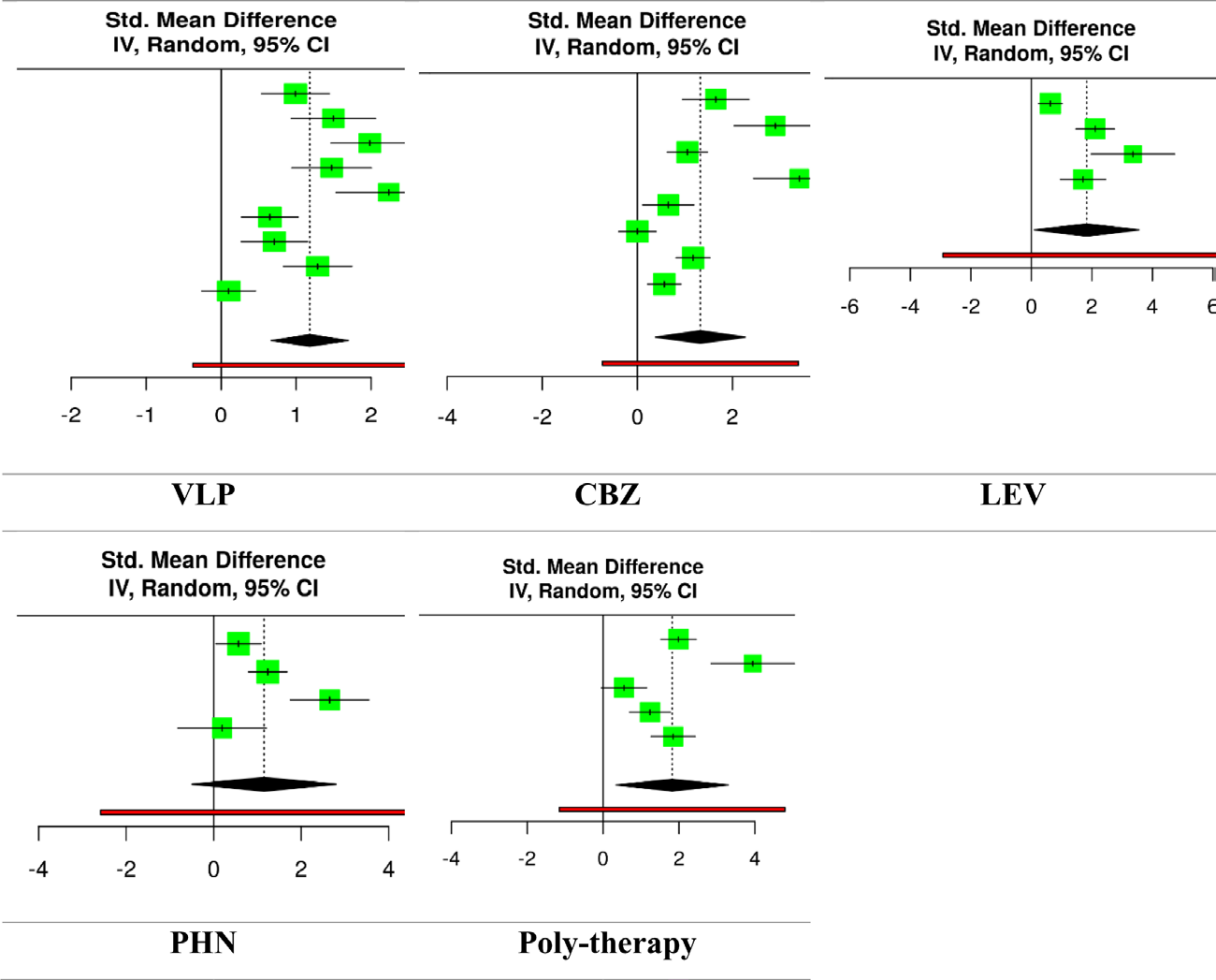
The forest plot of effect each anti-seizure medications on CIMT. CIMT = carotid intima–media thickness.

Statistical analysis of the data revealed that 676 individuals with epilepsy who underwent monotherapy with anticonvulsant drugs such as 280 on VPA,^[10,26–33]^ 233 on CBZ,^[10,29,30,32–36]^ 85 on LEV,^[10,26,28,29]^ and 78 PHN^[28,30,32,35]^ had higher CIMT compared to the 1067 participants in control group (VPA, standardized mean differences [SMD] = 1.18, 95% CI = 0.67–1.7, *P*-value < 0.001; CBZ, SMD = 1.33, 95% CI = 0.38–2.27, *P*-value < .001; LEV, SMD = 1.83, 95% CI = 0.1–3.56, *P*-value = .001; PHN, SMD = 1.15, 95% CI = −0.49 to 2.8, *P*-value = .007). This finding was also observed in our analysis of data related to 143 individuals underwent poly-therapy^[26,33,37–39]^: poly-therapy with anticonvulsant drugs was associated with a higher CIMT in patients who had epilepsy compared to the 192 individuals in control group (SMD = 1.82, 95% CI = 0.34–3.3, *P*-value < .001) (Fig. 2). However, the analysis demonstrated significant heterogeneity among all the above analyses (*P*-value < .001) (VPA, Q = 64.1, *I*^2^ = 87%; CBZ, Q = 81.33, *I*^2^ = 91%; LEV, Q = 28.24, *I*^2^ = 88.9%; PHN, Q = 19.8, *I*^2^ = 84%; poly-therapy, Q = 36.18, *I*^2^ = 88%).

Meta-regression analysis was conducted using available data from 5 studies^[30,33,37–39]^ that reported body mass index (BMI), lipid profiles, and treatment duration. The analysis revealed a significant positive association between BMI and CIMT (β = 0.12, *P* = .03), and between low-density lipoproteins (LDL-C) and CIMT (β = 0.18, *P* = .01). Treatment duration showed a non-significant trend (β = 0.05, *P* = .09). These findings suggest that metabolic factors may partially explain the observed heterogeneity. Moreover, subgroup analysis was not performed due to insufficient and inconsistently reported data across studies, particularly regarding ASM type, dosage, and participant characteristics, which limited the feasibility of meaningful stratification.

**Figure 3.**
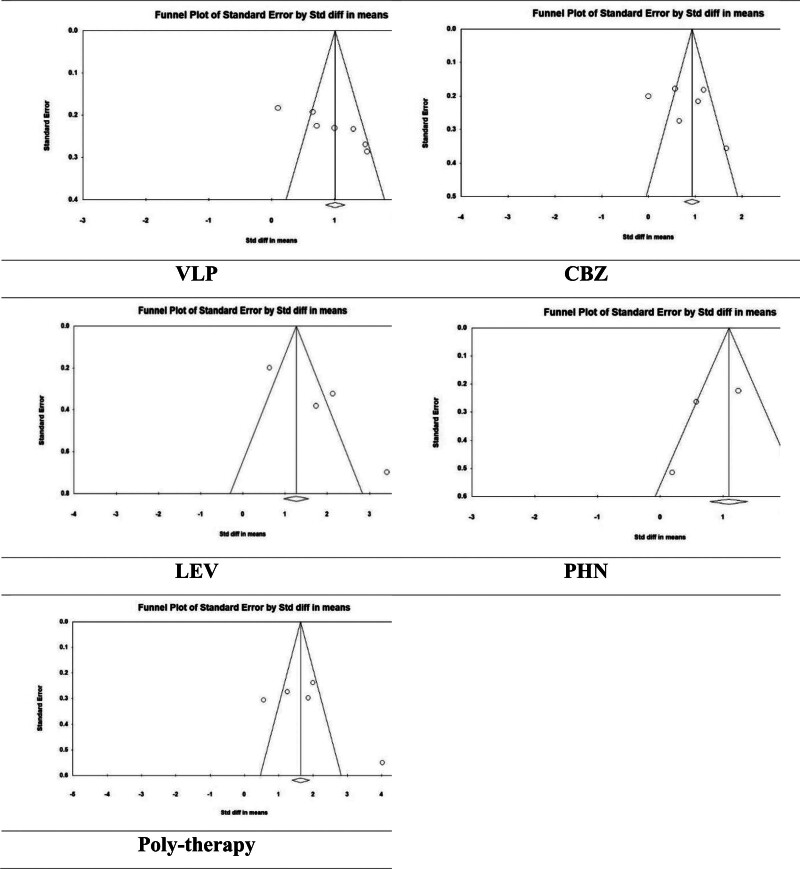
The publication bias status according to funnel plot asymmetry and Egger test.

In addition, based on funnel plot asymmetry and Eggers’ test, there was no potential publication bias in the evaluations except in the VPA and CBZ groups. (VPA, *P*-value = .003; CBZ, *P*-value = .04; LEV, *P*-value = .08; PHN, *P*-value = .82; and poly-therapy, *P*-value = .37) (Fig. 3). Given the substantial heterogeneity (*I*^2^ > 50%), we applied Duval and Tweedie Trim and Fill method using the random effects model for interpretation. For CBZ, no missing studies were imputed, and the pooled effect size remained unchanged (1.49861 [95% CI: 0.78060–2.21662]). For VPA, 1 study was imputed on the left side of the funnel plot, resulting in a slightly reduced pooled estimate from 1.31830 (95% CI: 0.94859–1.68801) to 1.21150 (95% CI: 0.83435–1.58865).

### 4.3. Risk of bias

**Figure 4.**
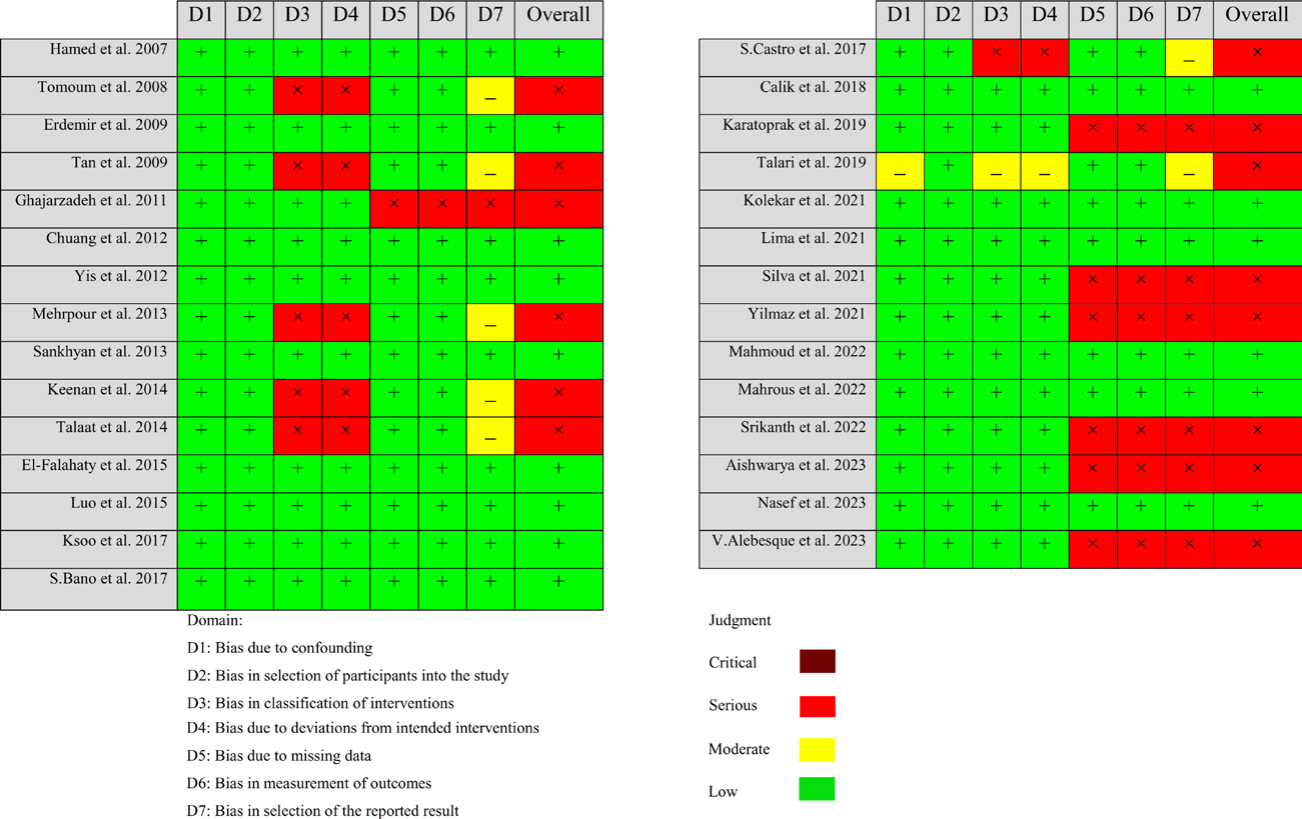
Risk assessment of bias using ROBINS-I for non-randomized studies in each study base on author’s judgment. ROBINS-I = risk of bias in non-randomized studies of interventions.

**Figure 5.**
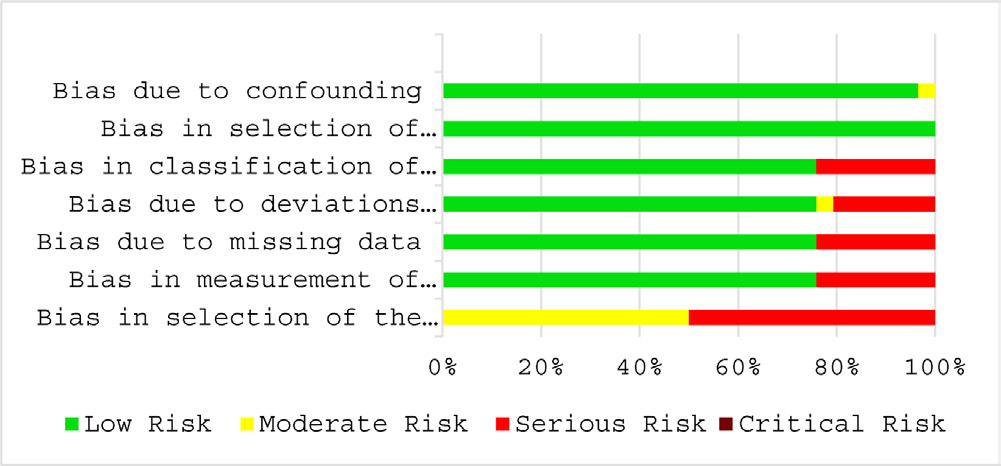
Risk of bias assessment of included based on subscales for all included studies based on authors’ judgment.

In the present systematic review and meta-analysis, we assessed the risk of bias and quality assessment based on ROBINS-I for non-randomized studies in each study. All included studies except one^[21]^ had a low risk confounding bias (D1). Six studies had high risk^[20,22–25,40]^ and 1 study had middle risk^[21]^ of bias in classification of interventions (D3) and deviations from intended interventions (D4). Bias due to missing data (D5) and bias in the measurement of outcomes (D6) among 7 studies was high.^[6,13–18]^ Also, 7 different studies had high risk^[6,13–18]^ and another 7 studies had middle risk^[19–25]^ of selection reporting bias (D7). All studies had a low risk of bias in selecting participants for the study (D2). In summary, the quality assessment based on the authors’ judgment depicted the low risk of bias for indicated studies (Figs. 4 and 5).

## 5. Discussion

In the current study we attempt to conduct a meta-analysis and systematic review on the effect of ASMs used by patients with epilepsy on CIMT that is measured by color doppler sonography. The duration of ASM therapy was varied among the included studies, but the mean range was reported from 6 months to 30.8 years among patients participating in our analysis. Patients included in our analysis reflected a diverse range of ages, from children to adults. Among the final included studies, 7 studies were excluded from the meta-analysis due to uncompleted reported data,^[6,13–17,41]^ and 7 studies were also excluded since the type of ASM and the result of exact CIMT for each medications were not clear.^[19–25]^ The results of our analysis reported that ASMs including Sodium Valproate, Phenytoin, Carbamazepine, and Levetiracetam can significantly increase CIMT among patients with epilepsy. Thus, the results of our study reflect an increased risk of cardiovascular disorders in patients with epilepsy using ASMs treatment. Also, the average overall bias of our included studies were categorized as a low risk and they had a good quality.

To assess publication bias, we applied the Duval and Tweedie Trim and Fill method. No missing studies were imputed for CBZ, and the pooled effect size remained unchanged, indicating minimal bias. For VPA, 1 study was imputed, slightly reducing the effect size, though the adjusted estimate remained consistent with the original, suggesting that any bias had limited impact on our conclusions.

To further validate the stability of our findings, we conducted a sensitivity analysis by sequentially excluding individual studies. The pooled effect sizes remained consistent, suggesting that the results are not driven by any single study and are robust across the included literature.

Previous studies demonstrated that patients with epilepsy who are on long-term treatment with ASMs may suffer from cardiovascular disorders, which can be reflected through atherosclerosis and increased CIMT in these patients.^[33,42]^ Several vascular disorders including arterial stiffness, increased CIMT, and increased IMT of bifurcation area were reported in patients with epilepsy who receive ASM therapy.^[43]^ Moreover, some studies reported higher rates of early death due to ischemic heart disease in patients with chronic epilepsy.^[44,45]^ A higher incidence of stroke in patients with chronic epilepsy was also reported through investigations.^[46]^ It is reported that ASM therapy in patients with epilepsy can cause hyperhomocysteinemia,^[47]^ weight gain,^[48]^ type 2 diabetes, hyperuricemia,^[49]^ and high levels of C-reactive protein (CRP), which in turn exacerbate the risk of cardiovascular disorders in these patients.^[50]^ These metabolic changes may promote vascular injury through oxidative stress and lipid peroxidation. Elevated homocysteine levels stimulate reactive oxygen species production, leading to LDL oxidation and endothelial dysfunction.^[51]^ Similarly, hyperuricemia enhances reactive oxygen species generation and contributes to vascular damage.^[52]^ Enzyme-inducing ASMs such as phenobarbital, phenytoin, and carbamazepine have been associated with elevated lipid profiles which may accelerate atherosclerosis in these patients.^[40,50]^

As mentioned, it is shown that ASMs can increase the risk of atherosclerosis in patients with epilepsy.^[53,54]^ Moreover, recent studies reported increased CIMT as a reliable marker for increased risk of atherosclerosis in patients with epilepsy using ASMs.^[55]^

VPA has been associated with metabolic disturbances such as weight gain,^[56]^ insulin resistance,^[57]^ and hyperlipidemia,^[58]^ which are established risk factors for atherosclerosis. Consistently, Lai et al, in their study, demonstrated that ASMs, especially Valproic acid, can significantly increase CIMT.^[53]^ However, some studies suggested no significant alterations in CIMT of children with epilepsy 1 year after treatment with Valproic acid.^[59]^ Moreover, increased CIMT was also reported in patients with epilepsy using Levetiracetam. Still, it is suggested that patients using Levetiracetam reflect a lower thickening rate of CIMT compared to patients taking other types of ASMs.^[29]^ In line with these findings, our meta-analysis revealed differences in SMDs across ASMs, with Levetiracetam showing a lower effect size on CIMT compared to Phenytoin. This variation may reflect differences in metabolic impact, enzyme-inducing properties, or treatment duration. These observations underscore the importance of individualized ASM selection and highlight the need for standardized reporting of dosage, comorbidities, and lifestyle factors to enable more robust meta-regression analyses.

Recent studies reported a significant increase in the thickness of common carotid IMT in patients using carbamazepine. These studies also reflected a significant correlation between the duration of carbamazepine therapy and right CIMT, left CIMT, and mean CIMT.^[32,60]^ Increased thickness of CIMT was also detected in patients with epilepsy using other ASMs including phenytoin and phenobarbital via B-mode ultrasonography.^[61]^ Chuang et al, in their study, reported a significant correlation between the duration of ASM treatment and CIMT in patients with epilepsy.^[30]^ Albeit, other studies reported that the duration of ASM therapy can not significantly impact the thickness of CIM in these patients.^[24,25,55]^

In addition, some studies demonstrated a significant correlation between CIMT and lipid profile in patients with epilepsy receiving ASMs.^[62]^ It is shown that enzyme-inducer ASMs such as phenobarbital, phenytoin, and carbamazepine accelerate the P450 cytochrome activity which in turn increases the synthesis of cholesterol in the liver.^[63]^ Thus, patients receiving enzyme-inducer ASMs reflect higher levels of total cholesterol, triglycerides (TG), and low-density lipoprotein cholesterol (LDL-C) than healthy people causing a higher risk of cardiovascular disorders in these patients.^[64]^ In addition, increased levels of Lipid profile (a) were shown in patients using Phenobarbital, Carbamazepine, and Sodium Valproate which can significantly exaggerate the atherosclerosis process in blood vessels.^[40]^ Some investigations indicated that ASMs which are enzyme-inducers can weaken the effect of statins in decreasing the level of LDL-C and thus fade the role of statins in avoiding atherosclerosis and cardiovascular impairments.^[65,66]^ Accordingly, in patients with epilepsy on enzyme-inducer ASM therapy the dosage of statins should increase to keep their efficiency in controlling the levels of total cholesterol and LDL-C.^[67]^

To further explore sources of heterogeneity, we performed a meta-regression using data from 5 studies^[30,33,37–39]^ that reported BMI, lipid profiles, and treatment duration. The analysis revealed significant associations between increased CIMT and both BMI and LDL-C levels, supporting the hypothesis that metabolic disturbances contribute to vascular changes in ASM-treated patients. Subgroup analysis based on ASM dosage, treatment duration, and patient characteristics was not feasible due to inconsistent and incomplete reporting across studies. These insights may inform personalized cardiovascular risk management strategies in epilepsy care, particularly for those on enzyme-inducing ASMs.

In addition to metabolic factors, some studies also assessed the effect of specific supplementations – including folate, vitamin B12, and vitamin B6 – on CIMT in this patient population. Most investigations revealed no significant alterations in CIMT among patients receiving folate supplementation.^[62,68,69]^ However, 1 study reported a significant decrease in CIMT after 18 months of folate supplementation^[70]^ and another found similar benefits following 1 year of combined folate, vitamin B12, and vitamin B6 supplementation in ASM-treated participants.^[71]^

Among the studies included in our meta-analysis, the majority employed cross-sectional designs and did not report baseline CIMT measurements prior to ASM initiation. This limits the ability to establish causality and raises the possibility of reverse causality, where preexisting vascular changes may influence ASM selection. However, 2 longitudinal studies^[53,59]^ did report baseline CIMT and demonstrated progressive thickening over time with ASM exposure, supporting a temporal association. These findings suggest a potential causal link, but further prospective cohort studies are needed to confirm directionality and control for confounding variables.

To enhance clinical applicability, we propose several practical recommendations. For patients receiving long-term VPA or CBZ therapy, we recommend baseline CIMT assessment followed by biennial monitoring. Risk mitigation strategies may include folic acid or B-vitamin supplementation to counteract ASM-induced hyperhomocysteinemia,^[70,71]^ regular screening of lipid profiles and homocysteine levels,^[62,64]^ and lifestyle interventions such as dietary modification and increased physical activity. For patients on enzyme-inducing ASMs, clinicians should consider more frequent lipid monitoring and potential statin dose adjustments to maintain cardiovascular protection.^[67]^ These strategies may help mitigate the vascular effects observed in ASM-treated patients and support more personalized cardiovascular risk management in clinical practice.

Our study has several limitations. First, some of the included studies had relatively small sample sizes, which may limit statistical power and reflect challenges in recruiting matched participants to control for potential confounding factors such as age, disease duration, sex, race, and socioeconomic status. Second, the cross-sectional design of most studies inherently limits the ability to establish causal relationships. Third, many participants had additional characteristics such as obesity, genetic predisposition, and lifestyle factors that may influence the effects of ASMs on CIMT and lipid profiles. Finally, inconsistent reporting of key variables such as drug dosage, treatment duration, and participant age restricted deeper stratified analyses.

## 6. Conclusion

In conclusion, our meta-analysis demonstrated that CIMT in patients treated with both mono- and poly-ASMs – including Sodium Valproate, Phenytoin, Carbamazepine, and Levetiracetam – was higher compared to the control group. These findings suggest that increased CIMT may reflect a higher cardiovascular risk in these patients, highlighting the need for careful long-term monitoring. Although we did not provide specific supplementation recommendations due to limited evidence, periodic CIMT assessment may be advisable for patients receiving these ASMs to better evaluate and manage cardiovascular risk.

## Author contributions

**Conceptualization:** Afshan Davari, Amir Reza Bahadori, Mehrdad Sheikhvatan, Abbas Tafakhori.

**Data curation:** Afshan Davari, Amir Reza Bahadori, Ali Mohammadi-Asl, Rasa Zafari.

**Formal analysis:** Afshan Davari.

**Methodology:** Amir Reza Bahadori, Ali Mohammadi-Asl.

**Supervision:** Amir Reza Bahadori, Sara Ranji, Abbas Tafakhori.

**Writing – original draft:** Afshan Davari, Amir Reza Bahadori, Ali Mohammadi-Asl, Rasa Zafari.

**Writing – review & editing:** Sara Ranji, Sajad Shafiee.

## Supplementary Material


