# Supplementary material for: Role of different anti-seizure medications on carotid intima–media thickness: A systematic review and meta-analysis
Source: Medicine (Baltimore). 2025 Nov 7;104(45):e45792. doi: 10.1097/MD.0000000000045792 (PMC12599644; doi:10.1097/MD.0000000000045792)
Supplement: Supplementary file 1 [file medi-104-e45792-s001.docx]

Search Strategy:

1. PubMed :
   ("Carotid Intima-Media Thickness"[Mesh] OR "Carotid Intima-Media Thickness"[tiab] OR ("CIMT"[tiab] NOT ("Constraint-induced movement therapy"[tiab] AND "Cancer Immunotherapy")) OR ("Ultrasonography, Carotid Arteries"[Mesh] AND (intima*[tiab] OR media*[tiab] OR intima-media[tiab]))) AND ("Epilepsy"[Mesh] OR epilep*[tiab] OR "seizure disorder*"[tiab] OR "Seizures"[Mesh] OR seizur*[tiab] OR convulsion*[tiab] OR convulsive[tiab] OR ictal[tiab] OR ictus[tiab])
2. Scopus:
   ( TITLE-ABS-KEY ( "Carotid Intima-Media Thickness" OR "Carotid Intima-Medial Thickness" OR "CIMT" OR "carotid intima" OR "carotid media" OR "carotid thickness" OR "carotid Ultrasound" OR "carotid sonography" ) AND TITLE-ABS-KEY ( "epilepsy" OR "epilepsies" OR "epileptic" OR "epilepsia" OR "epileptical" OR "seizure" OR "seizures" OR "convulsion" OR "convulsive" OR "ictal" OR "ictus" ) )
3. Web of Science:

("Carotid Intima-Media Thickness" OR "Carotid Intima-Media Thickness" OR (CIMT NOT ("Constraint-induced movement therapy" AND "Cancer Immunotherapy" )) OR ("Ultrasonography, Carotid Arteries" AND (intima* OR media* OR intima-media))) AND (Epilepsy OR epilep* OR "seizure disorder*" OR Seizures OR seizur* OR convulsion* OR convulsive OR ictal OR ictus)

1. Embase:

(exp "Carotid Intima-Media Thickness"/ OR "Carotid Intima-Media Thickness".tw. OR (CIMT.tw. NOT ("Constraint-induced movement therapy".tw. AND

"Cancer Immunotherapy")) OR (exp "Ultrasonography, Carotid Arteries"/ AND (intima*.tw. OR media*.tw. OR intimamedia.tw.))) AND (exp Epilepsy/ OR epilep*.tw. OR "seizuredisorder*".tw. OR exp Seizures/ OR seizur*.tw. OR convulsion*.tw. OR convulsive.tw. OR ictal.tw. OR ictus.tw.)

1. Cochrane Library:

([mh "Carotid Intima-Media Thickness"] OR "Carotid Intima-Media Thickness":ti,ab OR (CIMT:ti,ab NOT ("Constraint-induced movement therapy":ti,ab AND "Cancer Immunotherapy")) OR ([mh "Ultrasonography, Carotid Arteries"] AND (intima*:ti,ab OR media*:ti,ab OR intima-media:ti,ab))) AND ([mh Epilepsy] OR epilep*:ti,ab OR ("seizure" NEXT disorder*):ti,ab OR [mh Seizures] OR seizur*:ti,ab OR convulsion*:ti,ab OR convulsive:ti,ab OR ictal:ti,ab OR ictus:ti,ab)
